# Deep brain stimulation for dystonia

**DOI:** 10.1186/2047-9158-3-2

**Published:** 2014-01-21

**Authors:** Wei Hu, Matt Stead

**Affiliations:** 1 Department of Neurology, Mayo Clinic College of Medicine, 200 First Street SW, Rochester, MN 55901, USA

**Keywords:** Dystonia, Deep brain stimulation, Surgical outcomes, Neuromodulation, Globus pallidus

## Abstract

Deep brain stimulation (DBS) is an effective surgical treatment for medication-refractory movement disorders, and has been approved by the United States Food and Drug Administration for treatment of dystonia. The success of DBS in the treatment of dystonia depends on our understanding of the anatomy and physiology of this disorder and close collaboration between neurosurgeons, neurologists, clinical neurophysiologists, neuroradiologists and neuropsychologists. Currently, pallidal DBS is an established treatment option for medically refractive dystonia. This review is intended to provide a comprehensive review of the use of DBS for dystonia, focusing mainly on the surgical aspects, clinical outcome, MRI findings and side effects of DBS.

## Introduction

Dystonia is a movement disorder characterized by patterned directional and often sustained muscle contractions and causing twisting and repetitive movements or abnormal postures [1,2]. Dystonia is also commonly classified by three criteria: anatomical distribution (focal, segmental, or generalized); age of symptom onset (juvenile or adult onset) and etiology, primary, secondary, or symptomatic. Primary dystonias (especially generalized) are often hereditary and may be subdivided by genotype. Although the exact mechanism of dystonia is not well understood, several lines of evidence suggest that the basal ganglia play an important role in dystonia. Usually, medical treatments are unsatisfactory and limited by side effects. Although many focal cases and segmental dystonia may benefit from the treatment of botulinum toxin, some patients do not respond or become resistant to medical treatment later [1-4]. Moreover, botulinum toxin had no effect on the treatment of generalized dystonia [5]. DBS was first applied in cervical dystonia by Mundiner [6] in 1977. After that, two multicenter studies on bilateral Globus Pallidus pars Interna (GPi) DBS have demonstrated convincing clinical benefit on a large number of patients with primary generalized/segmental dystonia [7-9]. Accordingly, GPi DBS was approved by FDA in 2003 (as a humanitarian device exemption) for patients with chronic, medically intractable dystonia [10-13].

### Current application and outcome

All DBS candidates need to be evaluated by DBS neurologist for assessment of the severity of dystonia & disability level by appropriate rating scales, screening for genetic causes (particularly DYT-1 mutation) [14,15] , or secondary causes of dystonia. Moreover, cognitive and psychiatric assessments are also required as baseline measures. In our institute, the weekly Neuromodulation Committee meeting emphasizes multidisciplinary participation from Neurology, Neurosurgery, Neuropsychology, Psychiatry, Neuroradiology, Neurophysiology and other specialties. Potential dystonia patient candidates for DBS are discussed, and treatment options are considered. Didactic presentations covering current publications in neuromodulation is a regular component.

In order to be considered a DBS candidate, dystonic patients should be fairly disabled and have failed medical management. DYT-1 gene mutation test can also help us to predict the DBS outcome as it has been reported that DYT-1 positive patients had a better outcome [15,16]. However, the application of DBS in children with secondary dystonia is more controversial [17-19]. It has also been reported that duration of dystonia is negatively correlated with surgery prognosis; patients with shorter disease duration have more favorable postoperative outcomes. Thus, in order to prevent secondary orthopedic complications, DBS surgery needs to be considered earlier [15,20].

Recently, Moro E. et al. [21] nicely summarized several key points for DBS patients selection: briefly, young patients with primary dystonia and/or tardive dystonia are likely to have the good outcome from DBS surgery, however, regarding the patients with secondary dystonia, DBS should be carefully considered, due to lack of clinical effectiveness. Additionally, in patients with severe cervical dystonia, a cervical spine MRI is required to quantify the role of spinal degeneration in cervical pain [22], and to make sure whether or not if the spinal surgery is necessary before or after DBS [23]. Last but not the least the authors also recommend a detail psychiatric evaluation in patients with psychiatric history pre- and post-operatively.

### Optimal target selection

Once a patient is identified to be a suitable candidate for DBS, a target for the procedure has to be selected. Numerous studies have demonstrated that the GPi target can improve motor function and disability in primary dystonia [24-27]. The effects of GPi DBS on the cardinal symptoms of dystonia have been established in three randomized controlled clinical trials. Vidailhet, et al. reported a prospective, controlled, multicenter study in 2005 [8] and more recently in 2007 providing updated results [9]. Specifically, 22 severely impaired French patients with primary generalized dystonia were enrolled in the study and the efficacy and safety of pallidal DBS were evaluated with Burke-Fahn-Marsden Dystonia Rating Scale (BFMDRS) via a blinded review of videotaped sessions. The dystonia movement scores were significantly improved at the three-month evaluation. The overall quality of life was found to be improved at 1 year and maintained at 3 years. The adverse effects of lead fractures, infection and stimulated-related side-effects were reported in 5 patients and were resolved without permanent consequence. In 2006, Kupsch, et al. provided further evidence of benefit in a multicenter, randomized, sham-controlled, double-blind study [7]. Clinical results from 40 patients indicated substantial improvement in almost all movement symptoms (except speech and swallowing), disability level, and quality of life at 6 months post neurostimulation compared with baseline scores. Also, at 3 months, pallidal DBS neurostimulation was more effective than sham stimulation in patients with either primary generalized or segmental dystonia. Furthermore, GPi DBS has been demonstrated to provide long term motor and functional improvement in dystonia patients [7,27,28]. As reported in a 2 year follow-up retrospective study of 30 patients with primary dystonia, DBS patients were found to have excellent outcomes in motor and disability scores [29,30]. Importantly, for DTY-1 positive patients, GPi DBS demonstrated clinical benefit as long as ten years, however, 8 out of 26 patients had an additional GPi DBS because of the worsening dystonic symptoms [31]. In addition, according to a recent literature based series of 44 patients, children and adolescents responded well to GPi-DBS with good tolerance [32].

A few anecdotal reports suggested that the symptom of dysphonia can also be improved in selected DBS cases [33]. However, problems of speech and swallowing may be more difficult to manage with DBS as assessed by BFMDRS [7,8,34]. Worsening of handwriting and stimulation induced parkinsonism have also been reported in DBS patients with cervical dystonia [23,35-37].

On the other hand, patients’ perceptions of life shift after DBS should also be considered. Undoubtedly, most DBS dystonia patients had an easier, more satisfying life with greater confidence. However, some patients reported that the “new life” after DBS was challenging and stressful, because of concerning about being dependent on the stimulator and dealing with interfering side effects from stimulation. Those patients would need better professional support [38].

Dystonia-choreoathetosis Cerebral palsy is a common cause of disability in both children and adults, and refractory to pharmaceutical treatment. A recent multicentre prospective pilot study of bilateral GPi-DBS in 13 patients identified that clinical movement score, functional disability, pain, and mental health-related quality of life were significantly improved one year after DBS. The optimum therapeutic target was thought be the posterolateroventral region of the GPi [36,39-41].

Clinically, the use of DBS is controversial in children with secondary dystonia [19,42,43]. However, several case reports indicated that GPi DBS was effective in focal segmental dystonia involved in neck, facial, trunk, upper and lower limbs [44-50]. Interestingly, the combined targets of ventral intermediate nucleus (Vim) plus GPi DBS significantly improved Myoclonus Dystonia, a rare form of movement disorder with epsilon-sarcoglycan gene mutations and prominent action myoclonus plus dystonia [51]. Recently, the subthalamic nucleus (STN) has also been reported to be useful target in dystonia patients [10,52,53], Sun et al. proposed that STN DBS could potentially have following advantages over GPi DBS: immediately symptomatic improvement after programming, the lower stimulation parameters with longer battery life; and finally, STN DBS results in better symptomatic control [53]. However, currently, the data is limited and there is a lack of head to head comparisons between STN and GPi stimulation for dystonia. In contrast, secondary dystonia had a less robust response to DBS intervention [54]. The presumed reasons are that secondary dystonia is a complex movement disorders with a combination of hyperkinetic and akinetic-rigid dystonia, the DBS targeted structure often has lesions, and the dystonia is a progressively pathological process [32,50]. Taken together, although GPi stimulation is currently being regarded as the main DBS target for primary dystonias, focal, and segmental dystonias with substantial clinical benefit, we should continue studying the potential benefit of other targets, particularly for secondary dystonia patients [27].

**Figure 1:**
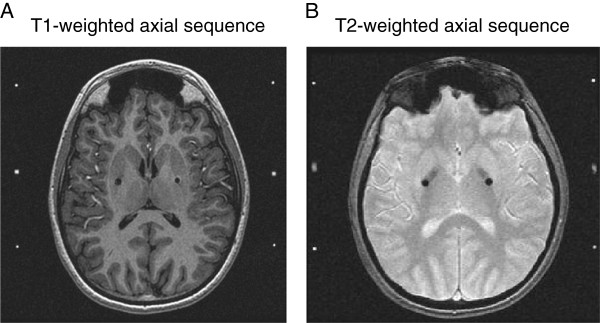
**Postoperative magnetic resonance images for the targets of bilateral Globus Pallidus Pars Interna from a representative patient. (A)** T1-weighted and **(B)** T2-weighted axial sequences are shown with the target highlighted with black dots.

**Figure 2:**
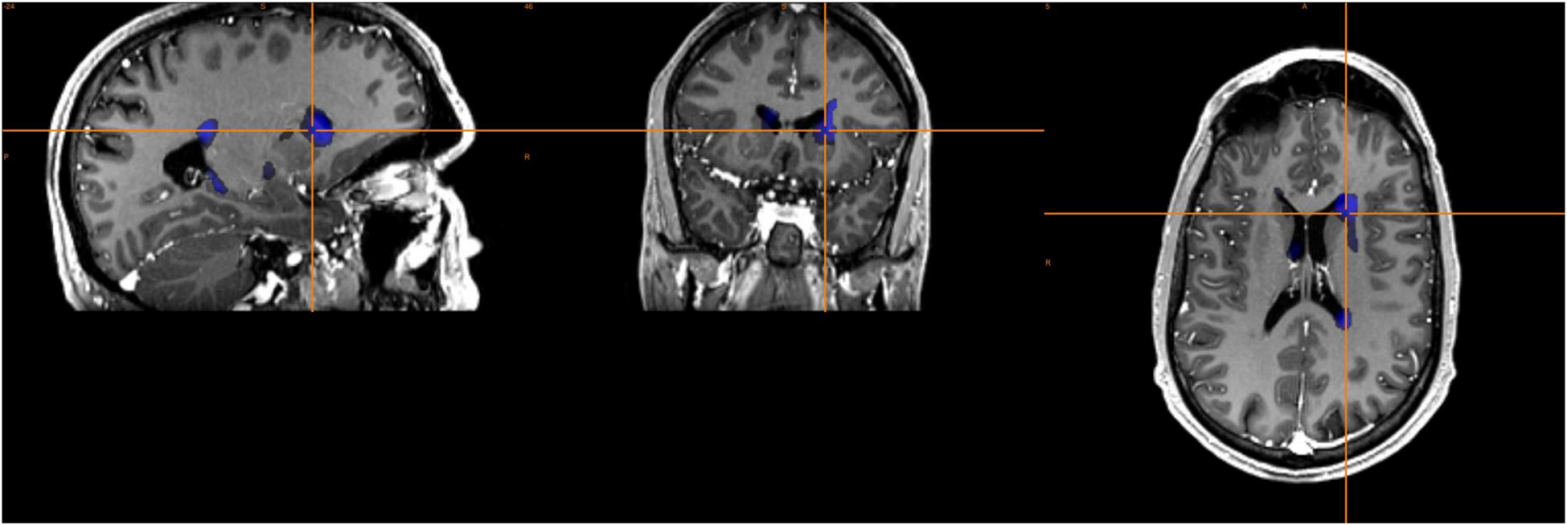
**Computer-aided subtraction SPECT imaging in a patient with severe dystonia.** The patient was 35 years old with long history of cervical dystonia and choreoathetotic movements. We obtained Technetium 99 m Neurolite SPECT scan of the brain in this patient with awake state and under anesthesia, respectively. Subtraction SPECT imaging had shown a hypermetabolic left caudate (blue), which is helpful for DBS target selection.

In our institute, intraoperative MRI has been routinely used in the DBS procedure. Specifically, post-implant imaging is supervised by an MR imaging physicist to maintain the specific absorption rate below the required level of 0.1 W/kg and always includes T1 magnetization-prepared rapid gradient echo and T2* gradient echo sequences with selected use of T2 fluid attenuated inversion recovery (FLAIR) and T2 fast spin echo (FSE). Recently, our DBS group demonstrated that intraoperative MR imaging during DBS lead placement can be performed safely to confirm lead placement prior to finalizing the surgery in all dystonia patients (Figure 1) [55]. Furthermore, since 1998, our department has used computer-aided subtraction ictal SPECT co-registered to MRI (SISCOM) to improve the clinical usefulness of SPECT in localizing the surgical epileptic seizure foci [56]. In 2010, we developed a statistical parametric mapping and MRI voxel-based method of analyzing ictal-interictal SPECT difference data (statistical ictal SPECT coregistered to MRI [STATISCOM]) and were able to determine whether the ictal-interictal subtraction difference is statistically different from the expected random variation between 2 SPECT studies and to further improve surgical accuracy and increases probability of seizure freedom for patients with epilepsy [57]. Based on the experiences obtained from epilepsy patients, recently, our DBS group had obtained Technetium 99 m Neurolite SPECT scan of the brain in the dystonia patient with awake state and under anesthesia, respectively. Then subtraction analysis was performed to determine whether the awake (dystonia)-sleep(non-dystonia) subtraction difference is statistically different and to further identify the target for DBS. We found that the location of hyperactivity (hypermetabolic) on the subtraction images in the brain could potentially help us to identify the optimal DBS target (Figure 2, unpublished data).

### Side effects

Overall, DBS patients tolerated the procedure fairly well [58], however, clinically, asymptomatic hemorrhage due to the DBS procedure may be difficult to be identified. Recently, our group found that intraoperative MRI is helpful in identifying acute changes involving intracranial hemorrhage and air during DBS surgery, given the fact that these findings are usually clinically silent and often resolve prior to follow-up imaging. We identified that selective use of T2 FLAIR and T2 fast spin echo (FSE) imaging can confirm the presence of air or hemorrhage and preclude the need for CT examinations [55]. On the other hand, hardware related complications including infection and electrode lead displacement, lead or extension fractures, may occur more frequently in dystonia as prominent axial movements cause more mechanical stress. These patients are more likely to complain of stimulation related adverse effects of speech problems, such as dysarthria and dysphonia It should be noted that, despite the fact that most studies indicate that bilateral GPi stimulation does not have significant adverse effect on mood or cognition in dystonia patients, several suicides cases have been reported in GPi DBS dystonia patients. All of them had depression at baseline and it is still unknown whether the suicide was related to the DBS stimulation [59]. As such, close monitoring should be warranted in these patients, given reports of postoperative suicide [60].

### Possible mechanism of action of DBS

Due to the unknown pathophysiology of dystonia [61], the knowledge about the mechanism about GPi DBS in dystonia is very limited. It appears that the time between the stimulation beginning and onset of symptom relief is longer (days to weeks) as compared to PD (minutes) [53]. It is plausible that GPi DBS increases output from the stimulated nucleus and activates surrounding fiber pathways, subsequently resulting in a complex pattern of excitatory and inhibitory effects that modulate the entire basal ganglia thalamocortical network. The stimulation-induced regularization of neuronal patterns prevents transmission of pathologic bursting and oscillatory activity within the network, resulting in improved processing of sensorimotor information and reduction of disease symptoms [62].

## Conclusion

DBS provides relief of the main symptoms of dystonia. It is plausible that the clinical benefits of DBS are due to the disruption of the pathological activity in the cotical-basal-gangalia-thalamic-cortical motor loop. Better understanding the mechanism of DBS will undoubtedly help more dystonia patients for long term benefit. With the advent of advanced neuroimaging, like SISCOM, STATISCOM, and 7-Tesla MRI, experienced DBS doctors will be able to optimal DBS target selection and identify the most suitable candidates for it [24]. In addition, new exciting developments of DBS technology is quickly evolving, currently, it is plausible for DBS programmer to have more flexibility in stimulation programming and increased battery duration. Increasing evidence will also help us to apply DBS in secondary dystonia patients and explore potential new optimal target for DBS [21]. Finally, the success of DBS treatment in the dystonia depends on our understanding of the anatomy and physiology of this disorder from basic scientists, and involvement of neurologists, neurosurgeons, neuroradiologists and neuropsychiatrists in outcome studies of DBS surgery.

## Competing interests

Dr. Matt Stead is the director of deep brain stimulation program at Mayo Clinic, and serves on the Medtronic Medical Advisory Board.

## Authors’ contributions

WH and MS have written the manuscript draft, Both authors read and approved the final manuscript.
